# Lamina-specific association between reduced mRNA levels of tyrosine kinase b and glutamate decarboxylase 67 in the orbitofrontal cortex in bipolar disorder: A possible reflective of defective connectivity in bipolar disorder

**DOI:** 10.1192/j.eurpsy.2023.471

**Published:** 2023-07-19

**Authors:** J. E. Park, J. Choi, S.-B. Jung, J.-C. Lee, I. B. Kim

**Affiliations:** ^1^Department of Psychiatry, Keyo Hospital, Uiwang; ^2^Department of Psychiatry, Hanyang University Medical College, Guri, Korea, Republic Of

## Abstract

**Introduction:**

Lamina-specific alterations of inhibitory circuitries have been considered the crucial pathogenesis of perceptual, cognitive and behavioral symptoms presented in schizophrenia and mood disorders. Especially, with emerging evidences indicating the close lamina-specific relationship between synaptic defects and γ-Aminobutyric acid (GABA)-related gene dysfunctions, it has been suggested the mRNA dysregulations of Tyrosine kinase B (TrkB) and Glutamate decarboxylase 67 (GAD67) could particularly be implicated in middle and deep layers of neocortex of patients with major psychiatric disorders.

**Objectives:**

Giving inquiries of whether defects of these mRNA levels in Orbitofrontal cortex (OFC) would be involved as lamina-specific patterns in individuals with schizophrenia and mood disorders.

**Methods:**

We examined mRNA levels of BDNF, TrkB and GAD67 in each OFC layer I through VI. We analyzed data from postmortem brain tissue of the Stanley Neuropathology Consortium Integrative Database (SNCID). SNCID consists of 15 subjects in each of four groups (schizophrenia, bipolar disorder, major depression without psychotic features, and unaffected controls). All groups were matched for age, sex, race, brain pH and post-mortem interval.

**Results:**

We found TrkB mRNA levels to be significantly reduced in layer VI in both groups with schizophrenia (25.8%) and bipolar disorder (35.7%) compared with controls. GAD67 mRNA levels were also significantly reduced in layer III and IV in patients with schizophrenia (23.4% and 22.7%, respectively) and bipolar disorder (31.2% and 24.9%, respectively) compared with controls. Individuals with major depression showed only trends toward decreased mRNA levels of GAD67 in layer III and IV and of TrkB in layer VI compared with controls. TrkB mRNA levels in layer VI were significantly correlated with GAD67 mRNA levels in layer III (**ρ**=0.581, p=0.037) and IV (**ρ**=0.857, p<0.001) in subjects with bipolar disorder, but not in those with schizophrenia. When analyzed with partial correlation controlling the effects of pH and PMI, significance of correlation remained only between GAD67 mRNA in layer IV and TrkB mRNA in layer VI in individuals with bipolar disorder (**ρ**=0.768, p=0.006).

**Conclusions:**

The resulting lamina-specific decreases in inhibitory tone across layers of OFC may contribute to the unrestrained irritability and violent behaviors in common shared by both patients with schizophrenia and bipolar disorder. Nonetheless, our findings indicate the obvious correlations between lamina-specifically altered TrkB and GAD67 mRNA levels in OFC might be a candidate for endophenotype of bipolar disorder.

**Disclosure of Interest:**

None Declared

